# Enhanced lipid production by *Rhodosporidium toruloides* using different fed-batch feeding strategies with lignocellulosic hydrolysate as the sole carbon source

**DOI:** 10.1186/s13068-016-0542-x

**Published:** 2016-06-23

**Authors:** Qiang Fei, Marykate O’Brien, Robert Nelson, Xiaowen Chen, Andrew Lowell, Nancy Dowe

**Affiliations:** School of Chemical Engineering and Technology, Xi’an Jiaotong University, Xi’an, 710049 China; National Bioenergy Center, National Renewable Energy Laboratory, 15013 Denver West Parkway, Golden, CO 80401 USA; KBI Biopharma, 2500 Central Ave, Boulder, CO 80301 USA

**Keywords:** Lignocellulosic hydrolysates, Lipid production, Fed-batch feeding strategy, *Rhodosporidium toruloides*, Automated online sugar control system

## Abstract

**Background:**

Industrial biotechnology that is able to provide environmentally friendly bio-based products has attracted more attention in replacing petroleum-based industries. Currently, most of the carbon sources used for fermentation-based bioprocesses are obtained from agricultural commodities that are used as foodstuff for human beings. Lignocellulose-derived sugars as the non-food, green, and sustainable alternative carbon sources have great potential to avoid this dilemma for producing the renewable, bio-based hydrocarbon fuel precursors, such as microbial lipid. Efficient bioconversion of lignocellulose-based sugars into lipids is one of the critical parameters for industrial application. Therefore, the fed-batch cultivation, which is a common method used in industrial applications, was investigated to achieve a high cell density culture along with high lipid yield and productivity.

**Results:**

In this study, several fed-batch strategies were explored to improve lipid production using lignocellulosic hydrolysates derived from corn stover. Compared to the batch culture giving a lipid yield of 0.19 g/g, the dissolved-oxygen-stat feeding mode increased the lipid yield to 0.23 g/g and the lipid productivity to 0.33 g/L/h. The pulse feeding mode further improved lipid productivity to 0.35 g/L/h and the yield to 0.24 g/g. However, the highest lipid yield (0.29 g/g) and productivity (0.4 g/L/h) were achieved using an automated online sugar control feeding mode, which gave a dry cell weight of 54 g/L and lipid content of 59 % (w/w). The major fatty acids of the lipid derived from lignocellulosic hydrolysates were predominately palmitic acid and oleic acid, which are similar to those of conventional oilseed plants.

**Conclusions:**

Our results suggest that the fed-batch feeding strategy can strongly influence the lipid production. The online sugar control feeding mode was the most appealing strategy for high cell density, lipid yield, and lipid productivity using lignocellulosic hydrolysates as the sole carbon source.

**Electronic supplementary material:**

The online version of this article (doi:10.1186/s13068-016-0542-x) contains supplementary material, which is available to authorized users.

## Background

Climate change and the increasing threat of global warming, which are most commonly caused by greenhouse gas (GHG) emissions, have drawn the attention of the world. Due to the GHG emissions from transportation, largely caused by the combustion of petroleum-based products [1], increased demand for renewable biofuels has gained tremendous attention in the last decade on the basis of their non-toxic, sustainable, and energy-efficient properties [2–4]. The current interest in renewable energy resources as alternatives to fossil fuels focuses around their capability to reduce the demand of crude oil and GHG emissions. Lipid is a potential feedstock for producing renewable hydrocarbon fuels [5]. However, using edible and industrial lipids derived from agricultural commodities results in competition with foodstuff necessary for the rising global population. Thus, microbial lipids from oleaginous microorganisms with high microbial lipid content in excess of 50 % (w/w) have been considered as alternatives to agricultural commodities for producing lipids and biofuels. *Rhodosporidium toruloides* is an industrially promising red oleaginous yeast, capable of converting pure glucose efficiently for microbial lipid production (Table 1) in terms of high lipid content, yield, and productivity [6–10].

**Table 1:** Comparison of lipid production by various oleaginous yeasts using different carbon substrates in batch and fed-batch cultures

Carbon substrate	Yeast strain	Culture mode	Lipid content^a^ (%)	Y_L/S_^b^ (g/g)	Pr_L_^c^ (g/L/h)	Refs.
Spent yeast	*Cryptococcus curvatus*	Repeated-batch	37	0.12	0.07	[14]
Sludge	*Cryptococcus curvatus*	Pulse-FB	23	–	0.09	[15]
Glycerol	*Cryptococcus curvatus*	DO-FB	25	0.11	0.59	[16]
Glycerol	*Cryptococcus curvatus*	Pulse-FB	53	0.20	0.06	[29]
Glycerol	*Yarrowia lipolytica*	Pulse-FB	31	0.08	0.18	[17]
Pure glucose	*Cryptococcus curvatus*	Pulse-FB	53	0.14	0.21	[30]
Pure glucose	*R. toruloides*	Pulse-FB	67	0.23	0.54	[6]
Pure glucose	*R. toruloides*	Pulse-FB	59	0.20	0.36	[7]
Lignocellulosic glucose	*Cryptococcus sp.*	Batch	60	0.13	0.05	[24]
Pure sugar mixture	*Rhodococcus opacus*	Pulse-FB	54	0.18	0.29	[31]
Pure sugar mixture	*R. toruloides*	Pulse-FB	58	0.07	0.15	[8]
Lignocellulosic sugar mixture	*Lipomyces kononenkoae*	Repeated-batch	59	0.16	0.18	[10]
Lignocellulosic sugar mixture	*Rhodococcus opacus*	Batch	54	0.13	0.13	[25]
Lignocellulosic sugar mixture	*R. toruloides*	Repeated-batch	61	0.16	0.13	[10]
Lignocellulosic sugar mixture	*R. toruloides*	DO-FB	56	0.14	0.33	[9]
Lignocellulosic sugar mixture	*R. toruloides*	Batch	59	0.19	0.28^d^	This work
Lignocellulosic sugar mixture	*R. toruloides*	DO-FB	60	0.23	0.33^d^	This work
Lignocellulosic sugar mixture	*R. toruloides*	Pulse-FB	62	0.24	0.35^d^	This work
Lignocellulosic sugar mixture	*R. toruloides*	Online-FB	59	0.29	0.40^d^	This work

^a^Lipid content, g lipid produced/g dry cell weight

^b^Y_L/S_: Lipid yield, g lipid produced/g carbon source consumed

^c^Pr_L_: Lipid productivity, g lipid produced/h/L

^d^Lipid productivity was calculated at 72 h when most of the sugars were consumed for lipid production

**Fig. 1:**
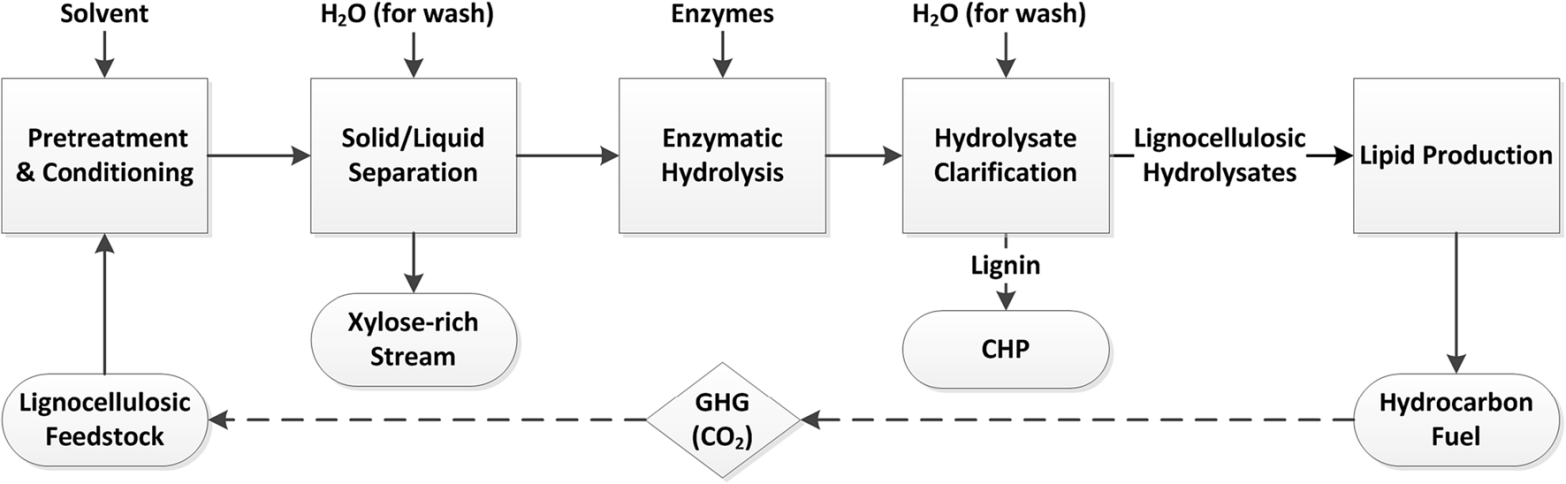
Simplified process flow diagram of lipid production from lignocellulosic feedstock. *CHP* represents combined heat and power

Although lipid production by oleaginous microorganisms provides a large-scale alternative method, the carbon source is another obstacle to the broader commercialization of the biofuel production. Currently, the fermentation-based bioprocess mainly relies on the carbon sources (glucose) derived from food (e.g., grain and corn). Recently published OECD-FAO Agricultural Outlook stated that more than 10 % of food was used for the production of biofuel globally [11]. Therefore, much effort has been focused on exploring new alternative carbon sources, including food waste [12, 13], wastewater [14], sludge [15], and glycerol [16, 17], among others. However, glucose appears to be the favorite carbon source for lipid production in terms of lipid yield and productivity compared with other alternative carbon substrates (Table 1). Lignocellulosic feedstocks, being the most abundant and sustainable biomass in the world [18, 19], can be used as the carbon substrates for fermentation processes after the appropriate pretreatment process. At the National Renewable Energy Laboratory (NREL), an integrated lignocellulosic ethanol process was successfully demonstrated at pilot scale using dilute acid pretreatment [20–22]. For microbial lipid production, we used a variation of the NREL lignocellulosic ethanol process. As shown in Fig. 1, the pretreated solids were first washed with water to separate the xylose-rich liquor and glucose-rich solid. The solids were converted to the lignocellulosic hydrolysates (glucose-rich stream) by enzymatic hydrolysis and then used as the carbon source for producing lipids. Hydrocarbon fuel (biofuel) derived from lignocellulosic feedstocks has the potential to be carbon neutral (as shown in Fig. 1), meaning that the loss of carbon dioxide (CO_2_) to the atmosphere caused by burning them is offset by the absorption of CO_2_ by the biofuel feedstocks when they are growing. Farell et al. [23] estimated that first generation biofuel (corn ethanol) can reduce GHG emissions by 18 %, while the second generation biofuel from lignocellulosic feedstocks is expected to reduce emissions by 88 % relative to petroleum-based fuels. The lignocellulosic hydrolysates derived from different biomass feedstocks have been investigated for lipid production in batch cultures [10, 24–27]. Although promising results were achieved from these studies, the lipid productivity was not sufficient for industrial application. It has been proposed that a minimum lipid productivity of 1 g/L/h is required to make microbial lipids economically competitive at industrial scale [28]. However, the lignocellulosic feedstock-derived carbon substrates can dramatically reduce raw material cost as well as the production cost to clear the critical demand of lipid productivity. Exploring other culture strategies may also help solve this obstacle leading to a better production performance.

Fed-batch cultivation is a batch culture mode with continuous or sequential feeding of the substrate, which is superior to batch cultivation in terms of eliminating substrate-associated growth inhibition and/or achieving high cell density culture (HCDC) [32]. Moreover, the culture operation of fed-batch is as simple and reliable as batch cultivation [33]. However, the fed-batch fermentation performance is highly dependent on the feeding strategy applied during the cultivation.

Several fed-batch strategies have been demonstrated in the literature for improving the fermentation performance of various desired products [9, 31, 33–35]. Simple indirect feedback control methods that couple substrate feeding with the measurement of pH or dissolved oxygen (DO) have been used for years, because they are relatively simple and inexpensive. However, the slow response of cells to pH or DO changes due to substrate utilization can cause over- or under-feeding, which could affect production [34]. In direct feedback control, monitoring the concentration of the residual growth-associated substrate in the fermentor is used for controlling feed. This can be accomplished manually (pulse feeding) or automatically (online control feeding) [33, 34]. Thus, to reduce the substrate-associated growth inhibition and achieve a high lipid yield and productivity, developing the fed-batch culture process is essential and necessary. Wiebe et al. [8] reported a fed-batch cultivation using a pulse feeding mode for producing lipids by *R. toruloides*. In that study, the lipid production was remarkably improved by intermittently feeding a pure glucose solution; lipid yield and productivity increased from 0.1 to 0.22 g/g and 0.08 to 0.21 g/L/h, respectively. As far as we know, however, very few studies have been conducted to date on developing different fed-batch feeding strategies for HCDC and efficient lipid production using lignocellulosic hydrolysates as the sole carbon source [9, 10].

In this paper, several effective high-cell-density fed-batch cultivation systems, including DO-stat feeding mode, pulse feeding mode, and online sugar control feeding mode, were developed to enhance lipid production by *R. toruloides* to achieve high lipid content, yield, and productivity. Finally, the fatty acid composition of microbial lipids produced in various fed-batch bioprocesses was analyzed and compared. Results from this study are useful for scaling up the microbial lipid production by *R. toruloides* for industrial applications.

## Results and discussion

### Lipid production in the batch cultivation using lignocellulosic hydrolysates as the sole carbon source

**Fig. 2:**
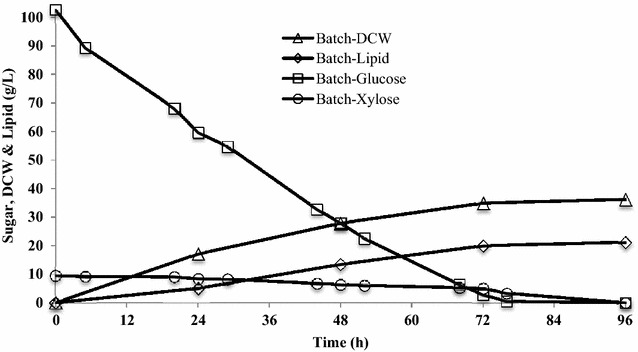
Cell growth and lipid production in batch cultures of *R. toruloides*. Total sugar consumption was 110 g/L (100 g/L glucose and 10 g/L xylose). Data points are the average of duplicate cultures. There was less than 5 % variation between each data point

To develop a suitable lipid production fermentation process, a batch culture of *R. toruloides* was performed to acquire baseline results for estimating the performance of lipid production in terms of lipid concentration, yield, and productivity. The batch cultivation was implemented using the aforementioned lignocellulosic hydrolysates with an initial concentration of 110 g/L, derived directly from the C6 cellulosic solids stream after enzymatic hydrolysis and hydrolysate clarification. The lignocellulosic hydrolysate sugar is composed of 95 % glucose and 5 % xylose. As shown in Fig. 2, dry cell weight (DCW) of 36.2 g/L was obtained from 96-h batch cultures after consuming all sugars (Fig. 2), which is similar to the previous report using pure glucose in the batch culture of *R. toruloides* giving a DCW of 37 g/L [8]. Our result indicates that the hydrolysates used in this study exhibited similar behavior as the pure sugar due to the processing step after pretreatment to produce the lignocellulosic sugars. The lipid content (50 %) and lipid productivity (0.19 g/L/h) from that work were lower than what we achieved from this study, which was 58.7 % and 0.28 g/L/h, respectively (Table 2). Our batch cultivation also gave a lipid yield of 0.19 g/g (g lipid produced/g carbon source consumed) on lignocellulosic hydrolysates, which was only 50 % of the theoretical yield (0.36 g/g [36]). This may be due to the inhibition effect of the high concentration of lignocellulosic hydrolysates over this batch cultivation and the nature of the batch cultivation. Li et al. observed that lipid yield dropped significantly when glucose concentration increased from 90 to 400 g/L [6]. It should be noted that the lipid yield from our batch cultures was higher than some previous literature (Table 1), which may be due to the difference in culture condition, microorganism, and carbon substrate used for lipid production. However, under the same culture conditions, a better lipid production can be achieved in fed-batch cultures compared with batch cultures [6, 8, 30, 37]. Therefore, several fed-batch feeding strategies were developed to obtain a better lipid production performance from lignocellulosic hydrolysates in terms of higher lipid yield and productivity.

**Table 2:** Lipid production by *R. toruloides* using lignocellulosic sugar mixture as the carbon source in batch, DO-stat fed-batch (DO-FB), pulse fed-batch (Pulse-FB), and online sugar monitoring fed-batch (Online-FB) cultures

Culture mode	Lipid content (%) (w/w)	Lipid yield (g/g)	Lipid productivity^a^ (g/L/h)
Batch	58.67 ± 2.28	0.19 ± 0.01	0.28 ± 0.01
DO-FB	59.81 ± 2.09	0.23 ± 0.01	0.33 ± 0.01
Pulse-FB	61.54 ± 2.87	0.24 ± 0.02	0.35 ± 0.01
Online-FB	58.76 ± 1.13	0.29 ± 0.01	0.40 ± 0.01

^a^Lipid productivity was calculated at 72 h when most of the sugars were consumed for lipid production

### DO-stat fed-batch cultivation for lipid production using lignocellulosic hydrolysates

The fed-batch culture mode proved to be one of the most efficient bioprocesses to reduce growth inhibition caused by the high concentration of the carbon source. The process of fed-batch cultivation had been applied for the production of various bioproducts, including, biopolyesters (PHAs), fatty acids, single cell proteins, etc. [32, 38, 39]. Three different fed-batch cultures of *R. toruloides* using DO-stat feeding, pulse feeding, and automated online feeding were carried out using C6-enriched lignocellulosic hydrolysates as the sole carbon sources.

**Fig. 3:**
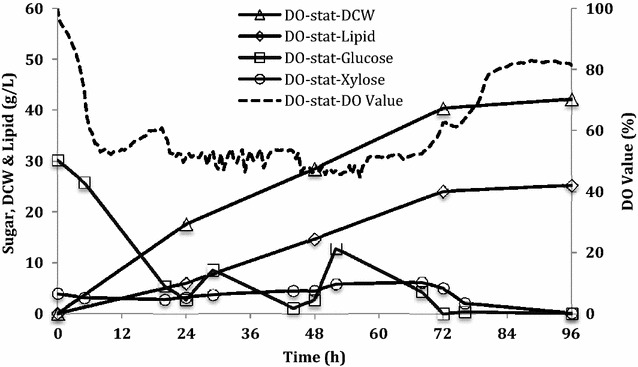
Cell growth and lipid production in DO-stat fed-batch (FB) cultures of *R. toruloides*. Total sugar consumption was 110 g/L (100 g/L glucose and 10 g/L xylose). Data points are the average of duplicate cultures. There was less than 5 % variation between each data point

DO-stat feeding strategy is an indirect feedback control that is the simplest and most inexpensive fed-batch culture system [40]. When sugar is supplied, the DO decreases rapidly due to the active metabolic pathways of cell growth and lipid production. Once the sugar is depleted, the DO begins to rise. Using these phenomena, a DO-stat fed-batch cultivation, in which DO was coupled with feeding sugars, was carried out. When DO reached more than 55 %, which was a preset value for this DO-stat fed-batch culture, the concentrated lignocellulosic hydrolysates were fed into fermentors automatically till the DO dropped to 50 % to prevent sugar depletion. Li et al. [6] found that the specific growth of *R. toruloides* decreased when glucose concentration was higher than 40 g/L. Therefore, the initial sugar concentration of lignocellulosic hydrolysates used for all the fed-batch cultures was 33 g/L hydrolysates (30 g/L glucose and 3 g/L xylose). The rest of the 77 g/L hydrolysate substrates were fed using different feeding strategies. The total sugar fed in fed-batch cultures corresponded to that in batch cultures. The time courses of DO, DCW, and sugar utilization in the DO-stat fed-batch cultivation are shown in Fig. 3. The abrupt increase and decrease of DO signals indicate the response to sugar exhaustion followed by addition of the lignocellulosic hydrolysates to the fermentors. Although the lipid content only improved by 1 % in this DO-stat fed-batch culture compared with our batch cultures, DCW and lipid productivity were increased more than 16 %, which corresponds to 42 g/L and 0.33 g/L/h, respectively (Fig. 3 and Table 2). Moreover, the lipid yield on lignocellulosic hydrolysates increased to 0.23 g/g (Table 2), which was a 21 % increase over what we observed in the batch cultivation. These results suggest that the DO-stat feeding strategy enhanced the efficiency of converting hydrolysates into lipid by *R. toruloides*. However, due to the nature of the indirect feedback control, the sugar concentration cannot be controlled precisely, which resulted in the under-feeding issue as can be seen at 24 and 44 h during this fed-batch cultivation. The sugar depletion over the cultivation may have an influence on both cell growth and lipid production [6], suggesting that better lipid production could be expected after solving this issue. Thus, developing the direct sugar control feeding strategy, which monitors the concentration of the residual substrate during the cultivation, is essential and necessary.

### Pulse feeding fed-batch cultivation for lipid production using lignocellulosic hydrolysates

**Fig. 4:**
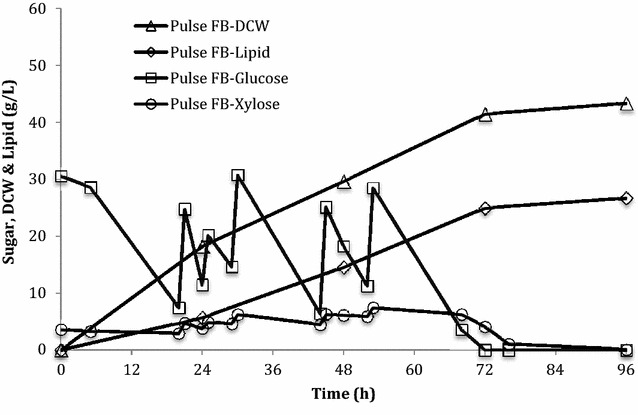
Cell growth and lipid production in pulse fed-batch (FB) cultures of *R. toruloides*. Total sugar consumption was 110 g/L (100 g/L glucose and 10 g/L xylose). Data points are the average of duplicate cultures. There was less than 5 % variation between each data point

The direct sugar feedback control is one of the most efficient feeding strategies to control commercial-scale bioprocesses [34, 41]. By measuring the sugar concentration either offline or online, the under-feeding of sugar during the cultivation can be prevented. Due to the growth inhibition effect when glucose concentration is greater than 40 g/L [6, 7], the sugar concentration in the pulse feeding fed-batch cultures was controlled below 30 g/L by manually feeding the concentrated lignocellulosic hydrolysates as shown in Fig. 4. The lignocellulosic hydrolysate feed was intermittently fed five times over 60 h to give a total sugar concentration of 110 g/L. A slightly higher DCW of 43 g/L and lipid content of 62 % were obtained in this pulse fed-batch culture (Fig. 4; Table 2). Because of the benefit of direct sugar feedback control, the sugar was maintained above 5 g/L throughout the entire cultivation, which improved the overall lipid yield and productivity to 0.24 g/g and 0.35 g/L/h, respectively (Table 2). Anschau et al. [42] applied a pulse feeding mode for lipid production by intermittently feeding concentrated sugar solution eight times in an 144 h cultivation. In this study, the lipid productivity in this pulse feeding fed-batch cultivation was improved by 20 % compared with the batch cultivation. Nevertheless, overfeeding appears to be another potential issue from this pulse feeding mode, even though a wide range of sugar concentration (5–30 g/L) did not affect the cell growth and lipid production compared to batch and DO-stat fed-batch cultures. It has been reported that glucose concentration has significant effects on both cell growth and lipid production in batch cultures of different oleaginous yeasts [6, 43]. However, overfeeding could be easily overcome by increasing sampling to maintain a desired level, but the intensive and extensive labor is not suitable or economical for an industrial-scale application. From this point of view, developing an online sugar control feeding mode, which can maintain the sugar at an optimal level automatically, will provide a great advantage over other fed-batch modes in terms of lipid production and scale-up for commercialization.

### Automated online sugar control fed-batch cultivation for lipid production using lignocellulosic hydrolysates

**Fig. 5:**
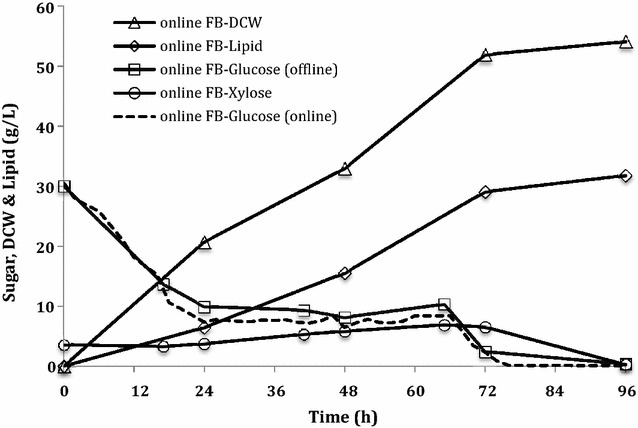
Cell growth and lipid production in online sugar monitoring fed-batch (FB) cultures of *R. toruloides*. Total sugar consumption was 110 g/L (100 g/L glucose and 10 g/L xylose). Data points are the average of duplicate cultures. There was less than 5 % variation between each data point

To maintain the desired sugar concentration during cultivation, an automated online sugar control feeding mode was developed using an automated cell-free sampling system coupled with an online sugar analyzer (details can be found in Methods and Materials). The automated sampling system will start feeding lignocellulosic hydrolysates based on the sugar concentration measured by the YSI and adjust the speed of the feeding pump to maintain a desired glucose concentration. It was suggested that maintaining a lower glucose concentration at 5 g/L in the cultivation of *R. toruloides* provided a 46 % increase in lipid productivity, compared with a 30 g/L glucose concentration [7]. Therefore, the glucose concentration was controlled at 10 g/L by this automated online sugar feeding system. As shown in Fig. 5, the glucose concentration measured by the online automated system via YSI (the dotted line) perfectly corresponded to the one measured offline by high performance liquid chromatography (HPLC) (the solid line: offline FB-Glucose). This automated system was able to control glucose around 10 ± 2 g/L, which provided a lipid yield of 0.29 g/g (Table 2) or 80 % of the theoretical yield (0.36 g/g [36]). The highest DCW of 54 g/L along with a lipid content of 59 % was achieved using this online sugar control fed-batch cultivation. Finally, the maximum lipid concentration of 32 g/L and lipid productivity of 0.4 g/L/h obtained from this fed-batch culture (Fig. 5 and Table 2) corresponded to a 52 and 42 % improvement, respectively, compared to our batch cultivation (Table 2). Slininger et al. [10] recently achieved lipid concentration of 30 g/L with lipid productivity of 0.22 g/L/h and lipid yield of 0.15 g/g in a two-stage process, which are unprecedented in any lignocellulosic hydrolysates. The results from our automated fed-batch system provided a higher lipid concentration, productivity, and yield compared with Slininger’s study [10] and exhibited the best lipid production performance achieved from lignocellulosic hydrolysates. This is likely because the *R. toruloides* cells converted the hydrolysate sugar more efficiently for cell mass generation and lipid production by controlling the glucose concentration around 10 g/L. Our result agrees with a previous report [44] that a higher sugar utilization rate and cell growth were achieved by maintaining glucose in a range of 12–17 g/L. This online sugar control fed-batch cultivation revealed that the highest cell density, lipid yield, and lipid productivity were obtained by maintaining an appropriate substrate level, indicating that this feeding mode efficiently converts lignocellulosic hydrolysates for lipid production by *R. toruloides*. Our findings suggest that using the proper culture mode, lignocellulosic sugar substrates can substitute for food-based sugars for producing lipid and biofuel, which will help to solve the food vs. fuel issue dramatically.

### Composition analysis of fatty acid produced by *R. toruloides* in various culture modes using lignocellulosic hydrolysates

The influence of different culture modes on fatty acid composition was investigated at the end of this study. The fatty acid profiles from four different culture modes are compared in Table 3. Although the lipid content from different culture modes is slightly different, fatty acid composition is very similar based upon the relative amount of total fatty acids. The fatty acids produced by *R. toruloides* in this study were predominantly 16 and 18 carbons (98 % of the total) and consisted of myristic acid (C14:0), palmitic acid (C16:0), stearic acid (C18:0), oleic acid (C18:1), linoleic acid (C18:2), and linolenic acid (C18:3). Our result agreed with previous reports that similar distributions of fatty acid were found in the cultivation of *R. toruloides* for lipid production [8, 45, 46]. These fatty acids are major precursors for biodiesel production [47]. The cetane number (CN), which measures the readiness of the diesel fuel to auto-ignite when injected into the engine [48, 49], is one of the most important properties to specify the quality of various biofuels used in a diesel engine, specifically the ignition quality that is one of the properties of biodiesel that is determined by the structure of the FAME component [50, 51]. According to an estimation equation [52], fatty acids produced with lignocellulosic hydrolysates in this work were expected to be in the upper 50 s, which are slightly lower than palm-based fatty acids, but higher than sesame and maize-based fatty acids (Table 3). Summarizing the result, fatty acids produced by *R. toruloides* using lignocellulosic hydrolysates as carbon substrates are promising raw materials for biodiesel production based upon the biofuel standards of the US and European Organizations, which require the minimal CN value to be about 50 [47].

**Table 3:** Comparison of fatty acid composition of lipids from *R. toruloides* and different feedstocks

Fatty acid source	Relative amount of total fatty acids (%)						CN^a^
	C14:0	C16:0	C18:0	C18:1n9	C18:2n6	C18:3n3	
Batch	1.3 ± 0.1	25.1 ± 1.1	10.1 ± 0.3	45.9 ± 2.1	10.5 ± 0.5	3.3 ± 0.1	57.7 ± 1.9
DO-FB	1.5 ± 0.1	26.5 ± 0.9	11.0 ± 0.2	46.4 ± 1.5	9.9 ± 0.3	3.0 ± 0.2	58.9 ± 2.1
Pulse-FB	1.3 ± 0.1	25.5 ± 1.2	10.9 ± 0.5	46.1 ± 2.2	10.1 ± 0.3	3.9 ± 0.2	58.2 ± 2.5
Online-FB	1.4 ± 0.1	26.8 ± 1.0	11.2 ± 0.5	45.8 ± 1.9	9.8 ± 0.2	4.1 ± 0.1	59.1 ± 2.2
Palm^b^	N.D.	44.8	4.6	38.9	9.5	0.4	61.4
Sesame^b^	N.D.	9.6	6.7	41.1	41.2	0.7	48.5
Maize^b^	N.D.	11.6	2.5	38.7	44.7	1.4	47.0

*N.D.* not detected

^a^CN: The cetane number was calculated as described by Gopinath et al. [52]: CN = 62.2 + 0.074X1 + 0.115X2 + 0.177X3 -0.103X4 − 0.279X5 − 0.366X6, where X1 to X6 indicates the weight percentages of methyl myristate (C14:0), palmitate (C16:0), palmitoleate (C16:1), stearate (C18:0), oleate (C18:1), linoleate (C18:2), and linolenate (C18:3) in lipids, respectively

^b^Data obtained from the literature [53]

## Conclusions

In this study, we successfully demonstrated the improvement in cell density, lipid yield, and lipid productivity from lignocellulosic hydrolysate by *R. toruloides* using an online sugar control feeding mode when compared with batch cultivation and other fed-batch feeding strategies. The online sugar control feeding mode produced a lipid yield of 0.29 g/g and a lipid productivity of 0.4 g/L/h, which correspond to a 52 and 42 % increase over batch cultivation, respectively. To the best of our knowledge and previous reports [10, 54], the lipid yield and productivity from this type of fermentation appears to be the highest reported yield achieved from lignocellulosic hydrolysates. Also, the online sugar control feeding mode yielded a maximum DCW and lipid concentration of 54 and 32 g/L, respectively. Two other fed-batch strategies tested also showed improvement over batch cultivation. The DO-FB and pulse-FB produced similar lipid production, resulting in a lipid yield of 0.23–0.24 g/L and lipid productivity of 0.33–0.35 g/L/h, which corresponds to a 20 % increase over batch cultivation. Our results indicate that the online sugar control feeding mode can greatly enhance the efficiency of converting the hydrolysates into lipids by *R. toruloides*. Furthermore, this is the first report comparing an automated fed-batch system with other typical fed-batch systems for bioconversion of lignocellulosic-based sugars for producing hydrocarbon fuel. The results achieved in this work provide valuable foundational knowledge that can be applied to future research to further optimize fed-batch cultivation for lipid production.

## Methods

### Microorganism, media, and chemicals

The red yeast *R. toruloides* DSMZ 4444 used in this study was purchased from Leibniz Institute DSMZ (German collection of microorganisms and cell cultures). The strain was maintained at −80 °C on yeast peptone dextrose (YPD) medium (20 g/L glucose, 20 g/L peptone, and 10 g/L yeast extract) with 20 % glycerol (v/v) and subcultured biweekly on YPD agar plate (15 % agar). The first seed culture medium (pH 5.2) consisted of 50 g/L glucose, 20 g/L peptone, and 10 g/L yeast extract. When the first seed culture reached the exponential growth phase, 10 % inocula were transferred to the second seed culture in the YNB culture medium (Cat# 1524-100, Sunrise Science Products Inc., San Diego, CA, USA) with 50 g/L glucose and 0.2X YP (2 g/L peptone, and 1 g/L yeast extract) for 2–3 days. The YNB culture medium contained the following ingredients (per liter): potassium phosphate (1000 mg), magnesium sulfate (500 mg), sodium chloride (100 mg), calcium chloride (100 mg/L), boric acid (0.5 mg), copper sulfate (0.04 mg), potassium iodide (0.1 mg), ferric chloride (0.2 mg), manganese sulfate (0.4 mg), sodium molybdate (0.2 mg), zinc sulfate (0.4 mg), and pH 5.2. All inocula (10 % of second seed culture) were resuspended with YNB culture medium before the inoculation for batch and fed-batch cultures. Unless otherwise stated, all chemicals were purchased from Sigma-Aldrich Co. LLC (St. Louis, MO, USA).

### Preparation of lignocellulosic hydrolysates

The simplified scheme for preparing lignocellulosic hydrolysates is shown in Fig. 1. Deacetylation and sulfuric acid impregnation were performed in the 1900-L dynamic impregnator tank. Dry corn stover was added to the tank along with a dilute sodium hydroxide solution (0.4 %wt/wt). The slurry was heated to 80 °C and held for 2 h, and then, the liquor was discharged. Water was then added to the tank along with a dilute sulfuric acid solution to achieve a 0.8 % acid concentration for pretreatment. After thoroughly mixing the acid and solids at room temperature for 2 h, the slurry was pumped to the Vincent screw press and dewatered to approximately 40 % solids. The horizontal pretreatment reactor (Metso Inc., Norcross, GA, USA) was preheated for 1 h using steam jackets and allowed to reach steady state at the desired temperature before the runs began. Feedstock was fed through the reactor at a rate of 25 dry kg/h. The reactor was operated at 160 °C with a residence time of 10 min. After leaving the reactor, the material was discharged into an atmospheric-pressure flash tank where it separated into a high solid pretreated slurry stream and volatile flash vent stream. The pretreated slurry was then discharged into 55-gal drums and stored under refrigeration. The pretreatment slurry was washed to remove the xylose-rich hydrolysate as well as residual acids and other downstream inhibitors. This was done by centrifugation in an 80-L Western States basket centrifuge (Fairfield, OH, USA). The slurry was loaded into the basket, and water was fed through the cake until the liquid exiting the centrifuge showed negligible amounts of sugars when tested on a YSI. The cake of washed solids was then collected and stored under refrigeration.

Enzymatic hydrolysis was performed using Cellic CTec2 provided by Novozymes (Franklinton, NC, USA). To maximize the amount of monomeric glucose, enzyme was loaded at 40 mg protein/g cellulose. The washed solids were neutralized using a commercial dough mixer to approximately pH 5.2 with 15 % (w/w) ammonium hydroxide. The neutralized pretreated biomass, enzyme, and sufficient water were combined to achieve a 20 % total solids loading and placed in custom-built 8-L baffled roller bottles. The bottles were then incubated in a custom-built, incubated roller bottle apparatus at 49 °C and 15 rpm for 7 days. The resulting enzyme hydrolysate was centrifuged in bottles using a Sorvall Lynx 6000 centrifuge (Thermo Scientific, Waltham, MA USA) to remove solids and then sterile-filtered using a Sartopure 2 XLG 0.8 + 0.2 µm capsule filter (Sartorius AG, Germany). The glucose-rich stream (lignocellulosic hydrolysates) was concentrated in Buchi rotary evaporators. Evaporation took place at 72.0 °C, 17 inches Hg vacuum, for 5–8 h to bring the overall sugar concentration in the lignocellulosic hydrolysate to approximately 550 g/L with a ratio of glucose:xylose = 10:1 measured by a HPLC.

### Culture condition

All seed cultures were grown in 250-ml Erlenmeyer flasks containing a 50-ml medium as specified above. The seed cultures were grown under 30 °C and 200 rpm in a rotary shaker. The exponentially growing cells were used for production cultures. All batch and fed-batch cultures were carried out in Sartorius BioStat Q-plus fermentors (Bohemia, NY, USA) with a 300 mL working volume in duplicate. Data points presented in this study are the average of duplicate cultures. The culture temperature was maintained at 30 °C, and pH was controlled at 5.2 with 4 M NaOH automatically by fermentors. The DO in the fed-batch cultures was maintained at 50 % of air saturation by adjusting the agitation speed from 300 to 700 rpm with an air flow rate of 0.4 volume per volume per minute (VVM). YNB culture medium with 0.2X YP was used for all production cultures. With the exception of the batch cultivation starting with 110 g/L lignocellulosic hydrolysates (100 g/L glucose and 10 g/L xylose), all fed-batch cultures were loaded with initial lignocellulosic hydrolysates of 33 g/L (30 g/L glucose and 3 g/L xylose) to reduce the lag phase due to less growth inhibition effect from low sugar concentration [6, 7]. Another 77 g/L of lignocellulosic hydrolysates (70 g/L glucose and 7 g/L xylose) was added during the cultivation via different feeding modes. The feeding solution was fed completely in 65 h in all fed-batch cultures, and all production cultures were terminated at 96 h when all sugars were consumed completely. The rapid increase in pH and DO values were observed after 72 h in all fed-batch cultures (see Additional files 1, 2, 3, 4, 5).

Three different feeding strategies were investigated to determine the best feeding mode in terms of cell density, lipid yield, and lipid productivity. In fed-batch cultures employing the DO-stat method, a rapid increase in the DO reading caused by the exhaustion of carbon substrates was used as an indicator for sugar feeding. The concentrated lignocellulosic hydrolysate was automatically fed with a peristaltic pump at different speeds when the DO burst to above 55 % until it dropped to below 50 %. The pulse feeding mode was carried out by feeding concentrated lignocellulosic hydrolysate manually during the cultivation after the residual glucose was lower than 10 g/L. The glucose concentration was maintained between 10 and 30 g/L for most of the culture time. An automated bioreactor sampling system was developed for the online sugar control feeding mode. The online YSI sugar analyzer (YSI 2950M, YSI Life Science, OH, USA) was coupled with an automated cell-free sampling system (Seg-Flow 1200, Flownamics Instruments Inc, Madison, WI, USA), which is capable of performing automated sampling (preset at every 2 h) and analysis during the fed-batch cultivation in response to the YSI sugar analysis data to control the substrate at an appropriate concentration. In this study, the concentrated lignocellulosic hydrolysate was automatically added in fermentors to maintain a glucose concentration of 10 g/L.

### Analytical methods

Cell growth was estimated by measuring OD at 600 nm. A sample of 10-mL culture broth was transferred to a preweighed centrifuge tube and centrifuged at 5000 rpm for 20 min. After rinsing the pellet once with deionized water, the pellet was centrifuged and frozen at −80 °C for 1–2 days, and then dried for 24 h in a lyophilizer for DCW measurement. Lipid content was measured as total FAME content after a whole biomass in situ transesterification procedure. In brief, 7–10 mg of lyophilized microbial biomass was homogenized with 0.2 mL of chloroform–methanol (2:1 v/v), followed by the transesterification procedure in situ with 0.3 mL of HCl–methanol (5 %, v/v) for 1 h at 85 °C. FAMEs were analyzed by gas chromatography–flame ionization detection (GC-FID) on an Agilent 6890N; DB-WAX-MS column (Agilent, Santa Clara, CA) with dimensions 30 m × 0.25 mm i.d. and 0.25 μm film thickness [55, 56]. Sugar concentrations were measured by a YSI sugar analyzer and/or HPLC (Santa Clara, CA. USA) based upon the NREL standard analytical procedure [57].

## Additional files

10.1186/s13068-016-0542-x Time course of pH during the DO-stat fed-batch cultures.
10.1186/s13068-016-0542-x Time course of pH during the pulse fed-batch (FB) cultures.
10.1186/s13068-016-0542-x Time course of dissolved oxygen (DO) value during the pulse fed-batch (FB) cultures.
10.1186/s13068-016-0542-x Time course of pH during the online fed-batch (FB) cultures.
10.1186/s13068-016-0542-x Time course of dissolved oxygen (DO) value during the online fed-batch (FB) cultures.

## Abbreviations

CHP: combined heat and power
C14:0: myristate
C16:0: palmitate
C16:1: palmitoleate
C18:0: stearate
C18:1: oleate
C18:2: inoleate
C18:3: linolenate
CN: cetane number
CO_2_: carbon dioxide
DCW: dry cell weight
DO: dissolved oxygen
rpm: rotations per minute
FAME: fatty acid methyl esters
FB: fed-batch
GHG: greenhouse gas
HCDC: high cell density culture
H_2_O: water
HPLC: high performance liquid chromatography
OD: optical density
NREL: National Renewable Energy Laboratory
VVM: volume per volume per minute
Y_L/S_: coefficient of lipid yield to sugar consumption
Pr_L_: volumetric lipid productivity
YPD: yeast extract, peptone extract and dextrose
