# Supplementary figures and images for: Enhanced lipid production by Rhodosporidium toruloides using different fed-batch feeding strategies with lignocellulosic hydrolysate as the sole carbon source
Source: Biotechnol Biofuels. 2016 Jun 23;9:130. doi: 10.1186/s13068-016-0542-x (PMC4918137; doi:10.1186/s13068-016-0542-x)

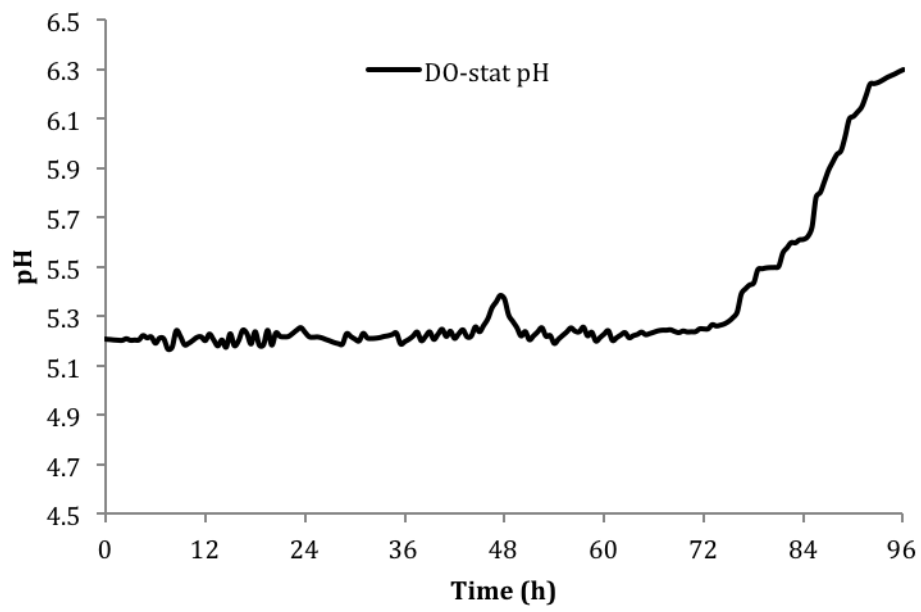

Figure S1: Time course of pH during the DO-stat fed-batch cultures.

Supplement: Supplementary file 1 — 10.1186/s13068-016-0542-x Time course of pH during the DO-stat fed-batch cultures. [file 13068_2016_542_MOESM1_ESM.pdf]

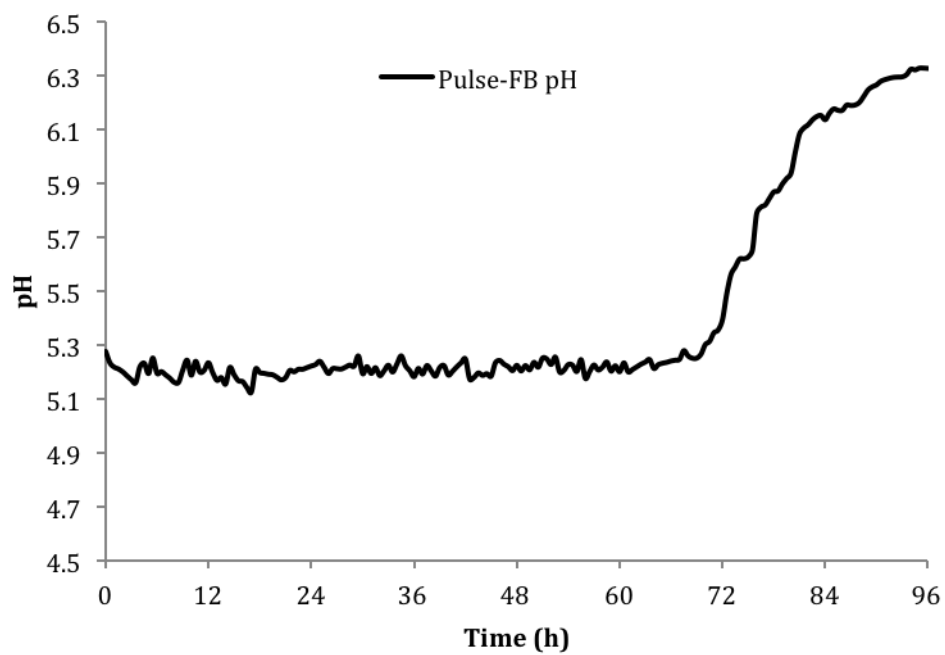

Figure S2: Time course of pH during the pulse fed-batch (FB) cultures.

Supplement: Supplementary file 2 — 10.1186/s13068-016-0542-x Time course of pH during the pulse fed-batch (FB) cultures. [file 13068_2016_542_MOESM2_ESM.pdf]

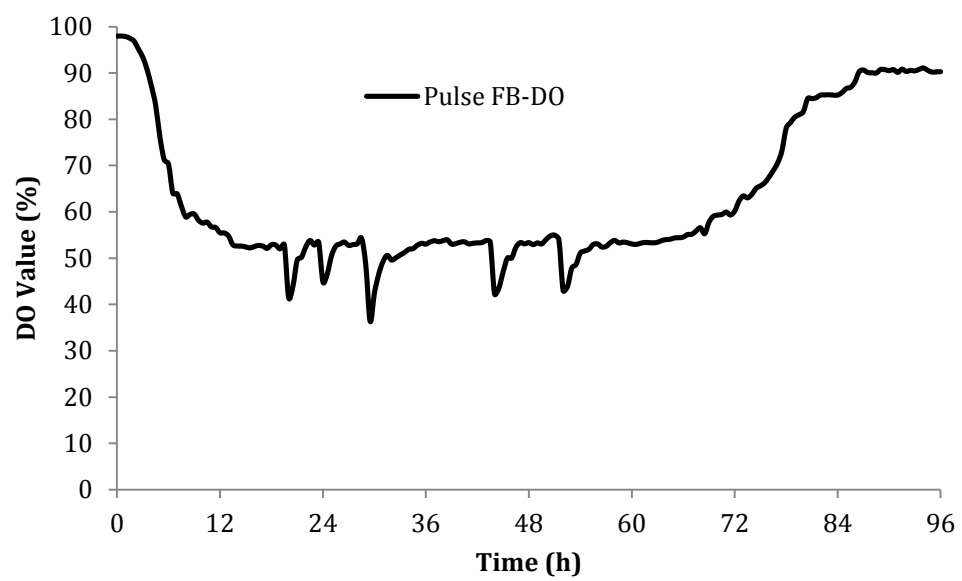

Figure S3: Time course of dissolved oxygen (DO) value during the pulse fed-batch (FB) cultures.

Supplement: Supplementary file 3 — 10.1186/s13068-016-0542-x Time course of dissolved oxygen (DO) value during the pulse fed-batch (FB) cultures. [file 13068_2016_542_MOESM3_ESM.pdf]

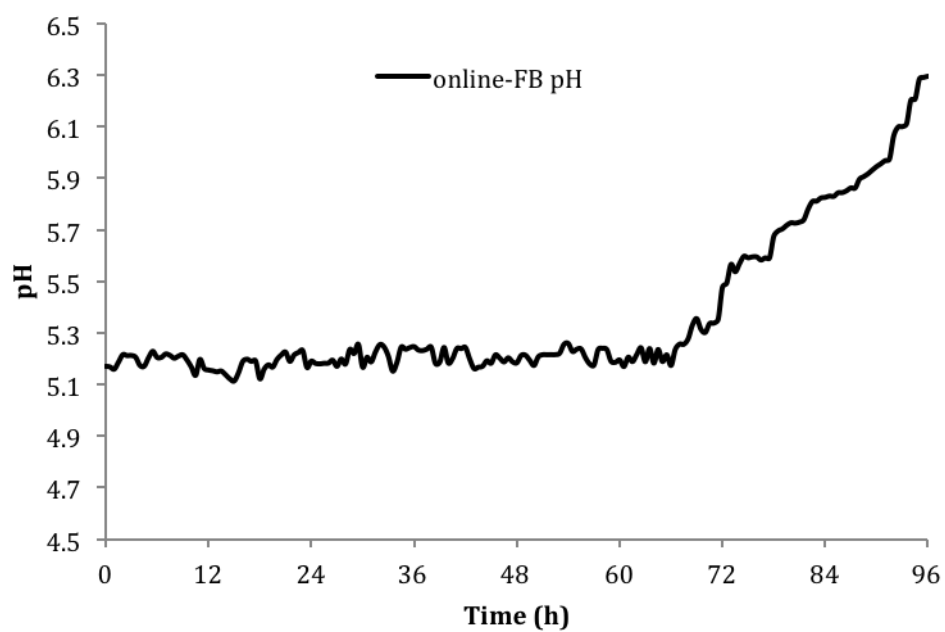

Figure S4: Time course of pH during the online fed-batch (FB) cultures.

Supplement: Supplementary file 4 — 10.1186/s13068-016-0542-x Time course of pH during the online fed-batch (FB) cultures. [file 13068_2016_542_MOESM4_ESM.pdf]

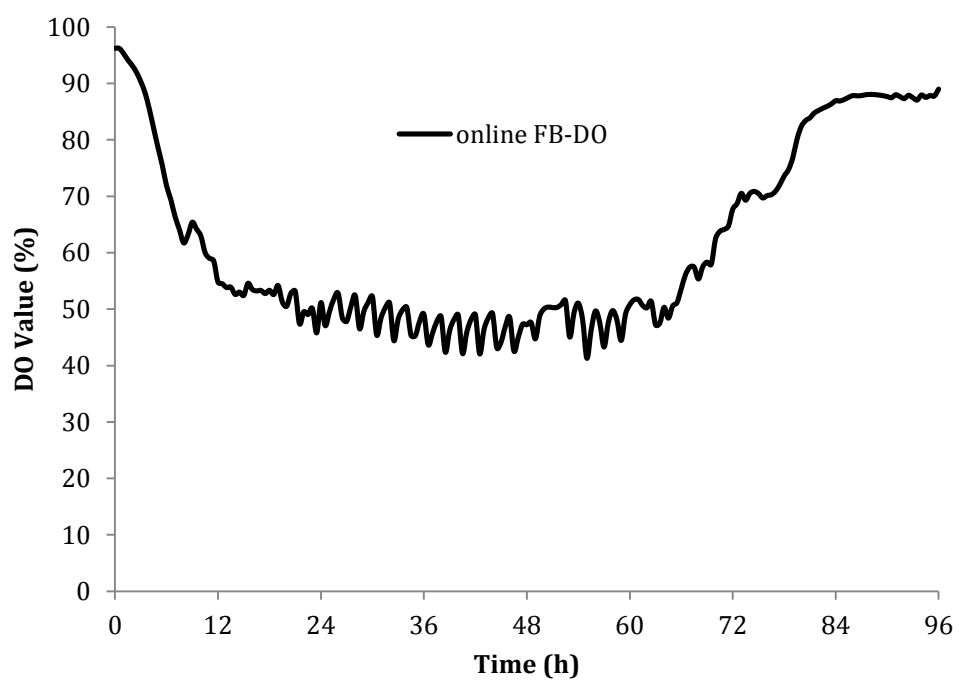

Figure S5: Time course of dissolved oxygen (DO) value during the online fed-batch (FB) cultures.

Supplement: Supplementary file 5 — 10.1186/s13068-016-0542-x Time course of dissolved oxygen (DO) value during the online fed-batch (FB) cultures. [file 13068_2016_542_MOESM5_ESM.pdf]
